# Early prehospital mechanical cardiopulmonary resuscitation use for out-of-hospital cardiac arrest: an observational study

**DOI:** 10.1186/s12873-024-01115-6

**Published:** 2024-10-19

**Authors:** Ying-Kuo Liu, Liang-Fu Chen, Szu-Wei Huang, Shih-Chan Hsu, Chin-Wang Hsu, Jen-Tang Sun, Shu-Hui Chang

**Affiliations:** 1 Department of Emergency Medicine, Wan Fang Hospital, Taipei Medical University, Taipei City, Taiwan; 2 Department of Medicine, Taipei Medical University, Taipei City, Taiwan; 3 New Taipei City Fire Department, New Taipei City, Taiwan; 4 Department of Pediatrics, Wan Fang Hospital, Taipei Medical University, Taipei, Taiwan; 5 Department of Emergency Medicine, Far Eastern Memorial Hospital, New Taipei City, Taiwan; 6 Department of Public Health, National Taiwan University, No. 17, Xuzhou Rd., Zhongzheng Dist., Taipei City (100), Taiwan

**Keywords:** Out-of-hospital cardiac arrest, Mechanical cardiopulmonary resuscitation, Emergency Medical System, Paramedics, Return of spontaneous circulation

## Abstract

**Background:**

The use of mechanical cardiopulmonary resuscitation device has been very prevalent in out-of-hospital cardiac arrest rescue. This study aimed to investigate whether the timing of mechanical cardiopulmonary resuscitation device set-up correlated with the the outcome of cardiac arrest patients.

**Methods:**

We retrospectively reviewed adult nontrauma cardiac arrest cases in New Taipei City, Taiwan, from January to December 2022. Demographic data, intervention-related factors, and the time variables of mechanical cardiopulmonary resuscitation were collected. The outcomes included the return of spontaneous circulation and 24-hour survival. We compared patients who achieved spontaneous circulation and those who did not with univariate and multivariable regression analyses.

**Results:**

In total, 1680 patients who received mechanical cardiopulmonary resuscitation were included in the analysis. Reducing the time interval from manual chest compression initiation to device setup was independently associated with the return of spontaneous circulation and 24-hour survival, especially in the subgroup of patients of initial shockable rhythm. Receiver operating characteristic analysis revealed that the outcome of patients with an initial shockable rhythm could be predicted by the mechanical cardiopulmonary resuscitation setup time, with areas under the curve of 60.8% and 63.9% for ROSC and 24-hour survival, respectively. The cutoff point was 395.5 s for patients with an initial shockable rhythm.

**Conclusion:**

A positive correlation was found between early mechanical cardiopulmonary resuscitation intervention and the outcomes of out-of-hospital cardiac arrest patients. The time between manual chest compression and device setup could predict the return of spontaneous circulation and 24-hour survival in the subgroup of patients with initially shockable rhythm with the optimal cutoff point at 395.5 s.

## Background

Out-of-hospital cardiac arrest (OHCA) is a major health issue that impacts countless families. Approximately 424,000 cardiac arrests are estimated to occur each year in the USA [1], 343,000 in Europe [2] and 19,344 in Taiwan in 2022 [3]. How to achieve better outcomes, including return of spontaneous circulation (ROSC) and survival to discharge with favorable neurological outcomes, is a topic of global concern for every emergent medical service (EMS) system. Although a variety of factors may influence outcomes, the chain of survival promoted by the American Heart Association (AHA) is the widely accepted concept for improving the rate of ROSC in OHCA patients [4]. This chain encompasses early recognition of cardiac arrest, activation of the EMS, high-quality cardiopulmonary resuscitation (CPR), rapid defibrillation if necessary, postcardiac arrest care and further recovery programs.

Metrics of high-quality CPR include the chest compression fraction (CCF), which is the cumulative time spent providing chest compressions divided by the total time taken for the entire resuscitation, should be over 80%, individual compression interruption less than 10 s, compression rate between 100–120/min, and compression depth at least 5 cm in adults. However, the shortage of emergency medical technicians (EMTs) makes high-quality CPR challenging; other competing tasks, including defibrillation, establishing vascular access, and a definite airway, may also distract paramedics from maintaining quality. A previous study revealed that seven or more prehospital providers are associated with a greater chance of survival [5]. Another study revealed that a crew size of 4–5 paramedics results in the best CCF, teamwork performance, and a shortened duration of advanced life support intervention [6]. However, in most cities in Taiwan, the majority of first dispatched team in is consisted of only 2 EMTs. Whenever OHCA identified by dispatched center or at the scene, additional support team will be dispatced for help. Since arrival time of support is hard to estimate, most teams tended to start transporting the patient after essential first-aid tasks done. This may lead to markedly fewer members compared to previous studies. Hence, with shortages of manpower, providing or maintaining high-quality CPR unless an additional compression device is applied may be impossible.

Mechanical CPR devices (MCPRs) were invented in the 1960s to help medical personnel maintain high-quality CPR. There are two main types of MCPRs: piston-based and vest-based. Both serve a similar function and perform automatic compression at a fixed rate and depth, even when moving downstairs or in a moving vehicle [7, 8]. Since transporting patients in a building is a significant reason for suboptimal CCF and CPR interruption [9], MCPR seems indispensable in resuscitation while transferring patients from buildings to hospitals for further care.

The proportion of MCPR utilization varies among countries, ranging between 5% and 60% [10–12]. However, approximately 80% of OHCA patients in Taiwan receive MCPR, which may be explained by a shortage of ambulance manpower and difficulty transporting [9]. Such vast experience enables us to investigate more details, such as how long it takes to complete the setup and whether or how the length of the MCPR setup affects OHCA patients’ outcomes.

Compared with traditional manual CPR, MCPR does not improve the outcomes of sustained ROSC, 4-hour survival or up to 30 days and does not favor neurological outcomes once reported [13–16]. Considering the dilemma between transportation and outcomes, the AHA recommends that in situations where conventional manual CPR cannot be safely and reliably performed, such as insufficient manpower, a long duration of first aid, a moving ambulance, during transport, or even an air ambulance, MCPR is a reasonable choice [17].

Recently, a Singapore-based EMS study revealed that OHCA patients who received MCPR at the scene had better clinical outcomes than did those who received MCPR after boarding the ambulance [18]. Another study reported that early installation of MCPR is associated with better outcomes in the prehospital setting, but no definite suggested timing has been reported [19]. Another study revealed that cardiac arrest patients who received MCPR less than 4 min after manual compression had a higher 4-hour survival rate, but this was the case in the emergency department [20]. Considering the inevitable utilization of MCPR due to the shortage of paramedic staff and difficult transportation, no previous study has focused on determining the proper timing of the prehospital MCPR setup. This study aimed to determine the relationship between the MCPR setup and OHCA patient outcomes.

## Methods

New Taipei City covers an area of 2,052 square kilometers and has a population of approximately 4 million, which is the highest among all cities in Taiwan. The Fire Department of New Taipei City Government (NTFD) governs eight branches and is responsible for 20,000 emergency cases monthly. Every year, approximately 2000 OHCA cases occur, and patients are sent to hospitals for further care. Upon receiving an emergency call, the NTFD dispatches paramedics from the nearest branch to the site where the patient has collapsed.

Paramedics received training programs regularly and complied with the suggestion of advanced cardiac life support (ACLS) guidelines by the AHA. In every case, paramedics are instructed to wear body cameras to record the attendance process. Upon arrival at the scene, paramedics approach the patient and initiate manual CPR immediately after recognizing cardiac arrest. They then applied automated external defibrillators (AEDs) or physiological monitors to differentiate between shockable and nonshockable rhythms and delivered defibrillation if indicated. Intravenous routes were also used for drug administration. Bag-valve-mask ventilation was initially used to provide oxygen, and supraglottic airway (SGA) or endotracheal tube insertion was attempted as advance airways if possible. If the patient’s body size was suitable for MCPR application on the basis of crew estimation and did not achieve ROSC during the manual CPR period, paramedics would set up MCPR before transportation. Crews are encouraged to minimize CPR interruption and maximize the CCF during MCPR set up; therefore, compression is resumed after placing the backplate prior to deploying the upper part or whenever MCPR malfunctions. Depending on paramedics’ clinical judgment, the device can be applied at any proper time. The MCPR device is a LUCAS-3^®^ (Physio-Control Inc., Redmond, WA, USA) in all branches.

Considering the lack of protocol for termination of resuscitation (TOR) in Taiwan, only in cases in which the bodys reach corpse decay, rigor mortis, entiredly charred, headless, internal organs spillage along with cardiac arrest, will be declared deceased on scene. Patients who are ambiguous, uncertain and have no family present or agree to participate will receive full resuscitation. After they board an ambulance, OHCA patients are transported to the nearest hospitals that provide emergency medical care.

### Inclusion and exclusion criteria

The study included patients who received resuscitation by the NTFD EMS from January to December 2022. Considering that our study aims to focus on the timing of the MCPR setup, the exclusion criteria are as follows: age younger than 18 years, trauma as the presumed origin, MCPR malformation, extreme body size, receiving only manual chest compression during resuscitation, resuscitation ceased at the scene, and those in whom resuscitative efforts were suspended according to the family’s will not resuscitate.

### Data collection

The demographic information of patients, including age, sex, where arrest occurred, and comorbidity profiles, were documented by paramedics in the electronic patient care reporting (ePCR) system of the NTFD. The details of the resuscitation process, including response time, presence of bystander CPR, initial rhythm, airway management, defibrillation, and medication, were also documented.

The quality control (QC) team of the NTFD was recruited in 2019 to improve the quality of prehospital care. The team collected information from AED records and the ePCR system and watched videos of body cameras worn on dispatched members. After being reviewed by the QC team member, peer review was conducted before data integration and analysis. Owing to possible biases among viewers, the QC team held consensus conferences quarterly and underwent calibration projects yearly or whenever new personnel joined.

**Fig. 1:**
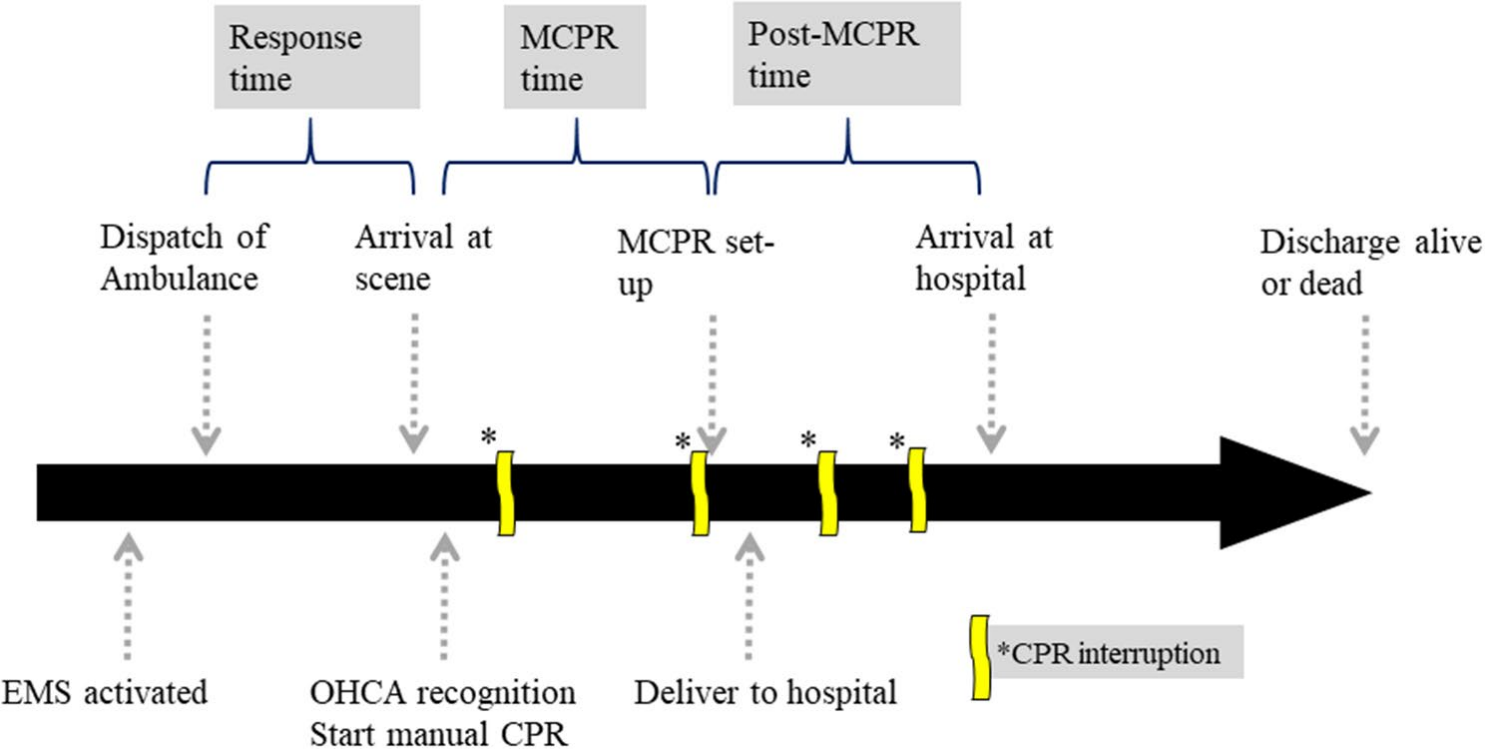
Timeline of the OHCA prehospital rescue process and relevant time intervals

We identified “pre-MCPR time” (defined as the interval between OHCA recognition and first MCPR compression, which may consist of manual compression, rhythm analysis, defibrillation, airway management, vascular assessment, medication administration, etc.), “post-MCPR time” (defined as the interval from MCPR initiation to handover of the patient to the hospital, which is mostly attributed to transportation), cumulative times of CPR interruption of more than 10 s and periods of CPR interruption due to the MCPR setup (Fig. 1). The NTFD will also keep track of those patients’ outcomes from the response hospital until discharge or death is announced.

### Outcome measures

Since our study focused on identifying possible factors between MCPR implementation and patient outcomes and long-term outcomes affected by postcardiac care in different hospitals, the ROSC is likely more strongly correlated with prehospital care and MCPR use. In our study, ROSC was defined as the maintenance of spontaneous circulation for at least 2 h, including in patients with prehospital ROSC. The presence of ROSC was defined as the primary outcome, whereas 24-hour survival was the secondary outcome.

### Statistical analysis

Demographic information and data regarding the prehospital management of ROSC and non-ROSC OHCA patients were compared. We used T-test for continuous variables. For categorical variables, chi-square test was conducted. Afterward, we conducted a linear regression to evaluate the associations between the time interval variables and outcomes. Multivariate logistic regression analysis was performed and included the variables for which the p value was < 0.05 in the univariate analysis. Considering time variables are the main goal of this article, we hope not to miss any potential factors, so all time variables with a p value less than 0.1 were included for further analysis. Besides, we utilized receiver operating characteristic (ROC) analysis to determine the optimal cutoff point of the MCPR time for predicting the ROSC status in OHCA patients, where the optimal cutoff point was defined as the point closest to the (0, 1) corner in the ROC plane. The data were analyzed via Rstudio software (Version 4.2.1, Boston, Posit Software).

### Ethical issues

The study was ethically approved by the Taipei Medical University-Joint Institutional Review Board (IRB No. N201909006). Each OHCA patient’s family member signed an informed consent after the resuscitation process in the ePCR system, allowing information about resuscitation to be analyzed and used for public health needs after identification.

## Results

### Patient population

**Fig. 2:**
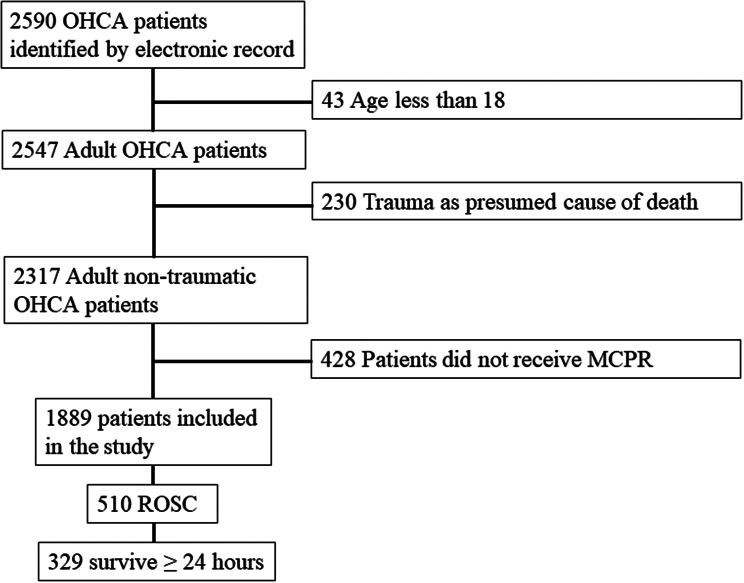
Participant flow

In summary, we identified 2590 OHCA patients from electronic records. After excluding pediatric patients (*n* = 43), patients with trauma as the presumed cause (*n* = 230), and patients who did not receive MCPR but only received manual chest compression (*n* = 428) (Table 1), 1889 patients were included in the study (Fig. 2). After these cases were reviewed, 209 patients received MCPR during resuscitation, but their pre-MCPR times were not reported due to video missing or poor video quality. Since other time intervals may be retrieved from other sources, including AED records or ePCR systems, we save those patients’ demographic data and available time interval data for study and define their MCPR time as not available during processing analysis. Among the 1680 patients included in the study, the median age was 66.8 years, and 32.9% were female. Only 13.0% of the population presented with an initial shockable rhythm, and 56.1% of them received bystander CPR. The sustained ROSC rate was 26.8%, the 24-hour survival rate was 16.7%, and the discharge rates of 3.8% and 2.3% of patients were cerebral performance categories 1 and 2, respectively.

**Table 1:** Comparison between OHCA patients with and without the MCPR setup

	MCPR (*N* = 1889)	No MCPR (*N* = 428)	*p* value
Demographic factors			
Age	68 (56–79)	71 (57–82)	0.010*
Sex, Female	622 (32.9)	194(45.3)	< 0.001*
Rhythm, shockable	256 (13.6)	51 (11.9)	0.039*
Bystander CPR	1047 (55.4)	193 (45.1)	< 0.001*
Place			0.003*
Home	1364(72.2)	275(64.3)	
Public place	219(11.6)	58(13.6)	
Others	306(16.2)	95(22.2)	
Response time(seconds)	406 (304-543.5)	391 (294.5–535)	0.079
Comorbidity			
Heart disease	474 (25.1)	99 (23.1)	0.431
Hypertension	638 (33.8)	127 (29.7)	0.116
Diabetes	486 (25.7)	89 (20.8)	0.038*
Malignancy	140 (7.4)	43 (10.0)	0.084
Cerebrovascular accident	112 (5.9)	27 (6.3)	0.853
Renal disease	190 (10.1)	30 (7.0)	0.064
Intervention-related factors			
Epinephrine	879 (46.5)	119 (27.8)	< 0.001*
Airway			< 0.001*
Supraglottic airway	1465(77.6)	210(49.1)	
Endotracheal tube	212(11.2)	39(9.1)	
Not done	212(11.2)	178(41.6)	
Interruption time(seconds)	83(40–141)	84(15–180)	0.712
Treatment outcomes			
ROSC	510(27.0)	175(40.9)	< 0.001*
24-H survival	329 (17.4)	129(30.1)	< 0.001*

### Primary outcome: ROSC

The demographic data of the OHCA patients in the ROSC and non-ROSC groups are presented in Table 2. OHCA patients who achieved ROSC were younger, had a greater proportion of initially shockable rhythm, and were more likely to be found in public places. Patients with ROSC tended to have shorter response times and were more likely to have comorbid heart disease than those without ROSC.

**Table 2:** Variables associated with the ROSC in OHCA patients

	ROSC(*N* = 510)	No ROSC(*N* = 1379)	*p* value
Demographic factors			
Age	66 (56–76)	68 (56–80)	0.02*
Sex, Female	185 (36.3)	437 (31.7)	0.07
Rhythm, shockable	105 (20.6)	151 (10.9)	< 0.001*
Bystander CPR	270 (52.9)	777 (56.3)	0.20
Place			0.001*
Home	348 (68.2)	1016 (73.7)	
Public place	82 (16.1)	137 (9.9)	
Others	80 (15.7)	226 (16.4)	
Response time(seconds)	384 (291–510)	415 (312–555)	< 0.001*
Comorbidity			
Heart disease	157 (30.8)	317 (23.0)	< 0.001*
Hypertension	184 (36.1)	454 (32.9)	0.22
Diabetes	133 (26.1)	353 (25.6)	0.88
Malignancy	40 (7.8)	100 (7.3)	0.74
Cerebrovascular accident	24 (4.7)	88 (6.4)	0.21
Renal disease	61 (12.0)	129 (9.4)	0.11
Intervention-related factors			
Epinephrine	254 (49.8)	616 (44.7)	0.05
Airway			0.001*
Supraglottic airway	421(82.5%)	1044(75.7%)	
Endotracheal tube	53(10.4%)	159(11.5%)	
Not done	36(7.1%)	176(12.8%)	
Interruption time(seconds)	67.41(35.75–133)	105.5(42–144)	0.01*
MCPR interruption time (seconds)	24.5(16–38)	25.74(15–40)	0.35
Pre-MCPR time (seconds)	442.5 (280.5-633.5)	470 (299.0-669.0)	0.06
Post-MCPR time (seconds)	875(638.5-1168.5)	938(698–1183.0)	0.01*

Footnote: CPR = Cardiopulmonary resuscitation, MCPR = Mechanical cardiopulmonary resuscitation, ROSC = Return of spontaneous circulation

*p value < 0.05

Patients in the ROSC group were more likely to receive defibrillation and SGA for advanced airway management. The cumulative interruption time and post-MCPR time were significantly shorter in the ROSC group. In contrast, the average pre-MCPR time was shorter in the ROSC group than in the non-ROSC group, but the difference was not statistically significant.

Table 3 shows the results of the univariate and multivariate logistic regression analyses. According to the multivariable analysis, OHCA patients who presented with an initially shockable rhythm (odds ratio (OR) = 1.52, 95% confidence interval (CI) = 1.08–2.13, *p* = 0.01), who were found at a public place (OR = 1.53, 95% CI = 1.07–2.19, *p* = 0.01), who had comorbid heart disease (OR = 1.51, 95% CI = 1.15–1.98, *p* = 0.003), and who had a shorter response time (OR = 0.97, 95% CI = 0.94–1.00, *p* = 0.04) had significantly greater odds of ROSC. For the resuscitation intervention, epinephrine injection (OR = 1.38, 95% CI 1.06–1.78, *p* = 0.01) and airway with endotracheal intubation (OR = 2.00, 95% CI 1.29–3.21, *p* = 0.003) were significantly associated with increased odds of ROSC. In addition, lower odds ratios of ROSC were observed per minute of increased pre-MCPR time (OR = 0.96, 95% CI 0.93–0.99, *p* = 0.01) and post-MCPR time (OR = 0.97, 95% CI 0.95–0.99, *p* = 0.002) during resuscitation.

**Table 3:** Univariate and multivariate predictors of ROSC

Parameter	OR	95% CI	*p* value	aOR	95% CI	*p* value
Demographic factors						
Age	0.99	0.99-1.00	0.04*	0.99	0.99- 1.00	0.16
Sex (female)	1.23	0.99–1.52	0.06			
Initial rhythm (shockable)	2.11	1.60–2.77	< 0.001*	**1.52**	**1.08–2.13**	0.01*
Bystander CPR	0.87	0.71–1.07	0.19			
Place						
Home	ref.			ref.		
Public place	0.57	0.43–0.77	< 0.001*	**1.53**	**1.07–2.19**	0.01*
others	0.97	0.73–1.29	0.81	0.93	0.67–1.30	0.69
Response time (minute)	0.96	0.93–0.98	< 0.001*	**0.97**	**0.94- 1.00**	0.04*
Comorbidity						
Heart disease	1.49	1.19–1.87	< 0.001*	**1.51**	**1.15–1.98**	0.003*
Hypertension	1.15	0.93–1.42	0.20			
Diabetes	1.02	0.81–1.29	0.83			
Malignancy	1.09	0.74–1.59	0.66			
Cerebrovascular accident	0.72	0.45–1.14	0.17			
Renal disease	1.31	0.95–1.81	0.10			
Intervention-related factors						
Epinephrine	1.23	1.00-1.51	0.04*	**1.38**	**1.06–1.78**	0.01*
Airway						
SGA	ref.			ref.		
Endotracheal tube	1.20	0.87–1.70	0.26	**2.00**	**1.29–3.21**	0.003*
Not done	1.96	1.36–2.90	< 0.001*	1.57	0.88–2.84	0.13
Interruption time (minute)	0.93	0.86-1.00	0.07	0.94	0.86–1.03	0.18
MCPR interruption time (minute)	0.91	0.72–1.14	0.46			
Pre-MCPR time (minute)	0.98	0.95-1.00	0.06	**0.96**	**0.93–0.99**	0.01*
Post-MCPR time (minute)	0.98	0.96-1.00	0.01*	**0.97**	**0.95–0.99**	0.002*

Footnote: aOR = adjusted odds ratio, CI = confidence interval, CPR = cardiopulmonary resuscitation, MCPR = mechanical cardiopulmonary resuscitation, Ref = reference, ROSC = return of spontaneous circulation

*p value < 0.05

### Secondary outcome: 24-hour survival

We compared demographic and intervention-related variables between those who survived 24 h after arrival at the emergency room and those who did not (Table 4). There were significant differences in age, initial rhythm, OHCA location distribution, response time, and heart disease as a comorbid condition between the two groups of OHCA patients. The average interruption time, pre-MCPR time, and post-MCPR time were significantly shorter in the 24-hour survival group.

**Table 4:** Variables associated with 24-hour survival in OHCA patients

	24-hour survival (*N* = 329)	No 24-hour survival (*N* = 1560)	*p* value
Demographic factors			
Age	64 (54–74)	68 (56.75-80)	< 0.001*
Sex, Female	115 (35.0)	507 (32.5)	0.43
Rhythm, shockable	86 (26.1)	170 (10.9)	< 0.001*
Bystander CPR	175 (53.2)	872 (55.9)	0.40
Place			0.01*
Home	221 (67.2)	1143 (73.3)	
Public place	54 (16.4)	165 (10.6)	
Others	54 (16.4)	252 (16.2)	
Response time(seconds)	377 (291–492)	411.5 (311–555)	< 0.001*
Comorbidity			
Heart disease	102 (31.0)	372 (23.8)	0.01*
Hypertension	118 (35.9)	520 (33.3)	0.41
Diabetes	84 (25.5)	402 (25.8)	0.98
Malignancy	23 (7.0)	117 (7.5)	0.84
Cerebrovascular accident	14 (4.3)	98 (6.3)	0.20
Renal disease	40 (12.2)	150 (9.6)	0.20
Intervention-related factors			
Epinephrine	157 (47.7)	713 (45.7)	0.55
Airway			0.06
Supraglottic airway	269 (81.8)	1196 (76.7)	
Endotracheal tube	38 (11.6)	174 (11.2)	
Not done	22 (6.7)	190 (12.2)	
Interruption time (seconds)	66(34–132)	85(41–143)	0.03*
MCPR interruption time (seconds)	25(15.75-37)	25(15–40)	0.50
Pre-MCPR time (seconds)	413 (265–618)	470 (299.0-670.0)	0.01*
Post-MCPR time (seconds)	854(630-1148.5)	933(695–1181.0)	0.01*

Footnote: CPR = Cardiopulmonary resuscitation, MCPR = Mechanical cardiopulmonary resuscitation, ROSC = Return of spontaneous circulation

*p value < 0.05

Univariate and multivariate regression analyses regarding 24-hour survival probabilities were also conducted (Table 5). We found that initially shockable rhythm (OR = 2.33, 95% CI 1.65–3.38, *p* < 0.001), younger age (OR = 0.99, 95% CI 0.98–0.99, *p* = 0.001), shorter response time (OR = 0.96, 95% CI 0.93–0.99, *p* = 0.02), and comorbidities of heart disease (OR = 1.55, 95% CI 1.14–2.09, *p* = 0.005) were significantly associated with higher odds of 24-hour survival. Airway management has reached significant levels regardless of whether endotracheal tube (OR = 2.55, 95% CI 1.49–4.65, *p* = 0.001) or bag-valve-mask ventilation (OR = 3.1, 95% CI 1.60–6.27, *p* = 0.001) is used. A lower odds ratio of 24-hour survival was observed per minute of increased pre-MCPR time (OR = 0.93, 95% CI 0.89–0.96, *p* < 0.001) and post-MCPR time (OR = 0.96, 95% CI 0.94–0.99, *p* = 0.001) during resuscitation.

**Table 5:** Univariate and multivariate predictors of 24-hour survival

Parameter	OR	95% CI	*p* value	aOR	95% CI	*p* value
Demographic factors						
Age	0.99	0.98–0.99	< 0.001*	**0.99**	**0.98–0.99**	0.001*
Sex (female)	0.90	0.70–1.15	0.39			
Initial rhythm (shockable)	2.89	2.15–3.87	< 0.001*	**2.33**	**1.65–3.38**	< 0.001*
Bystander CPR	0.90	0.71–1.74	0.36			
Place						
Home	ref.			ref.		
Public place	1.69	1.20–2.36	0.002*	1.31	0.88–1.93	0.27
others	1.11	0.79–1.53	0.54	1.05	0.72–1.52	0.05
Response time (minute)	0.95	0.92–0.98	< 0.001*	**0.96**	**0.93–0.99**	0.02*
Comorbidity						
Heart disease	1.44	1.10–1.86	0.007*	**1.55**	**1.14–2.09**	0.005*
Hypertension	1.12	0.87–1.43	0.38			
Diabetes	0.99	0.75–1.29	0.83			
Malignancy	0.93	0.57–1.46	0.77			
Cerebrovascular accident	0.67	0.36–1.15	0.15			
Renal disease	1.30	0.90–1.87	0.17			
Intervention-related factors						
Epinephrine	1.08	0.85–1.38	0.51			
Airway						
SGA	ref.			ref.		
Endotracheal tube	1.94	1.25–3.16	0.004*	**2.55**	**1.49–4.65**	0.001*
Not done	1.89	1.08–3.36	0.03*	**3.10**	**1.60–6.27**	0.001*
Interruption time (minute)	0.93	0.84–1.01	0.11			
MCPR interruption time (minute)	0.93	0.69–1.20	0.59			
Pre-MCPR time (minute)	0.96	0.93–0.99	0.01*	**0.93**	**0.89–0.96**	< 0.001*
Post-MCPR time (minute)	0.98	0.96-1.00	0.01*	**0.96**	**0.94–0.99**	0.001*

Footnote: aOR = adjusted odds ratio, CI = confidence interval, CPR = cardiopulmonary resuscitation, MCPR = mechanical cardiopulmonary resuscitation, Ref = reference, ROSC = return of spontaneous circulation

*p value < 0.05

### ROC analysis

**Fig. 3:**
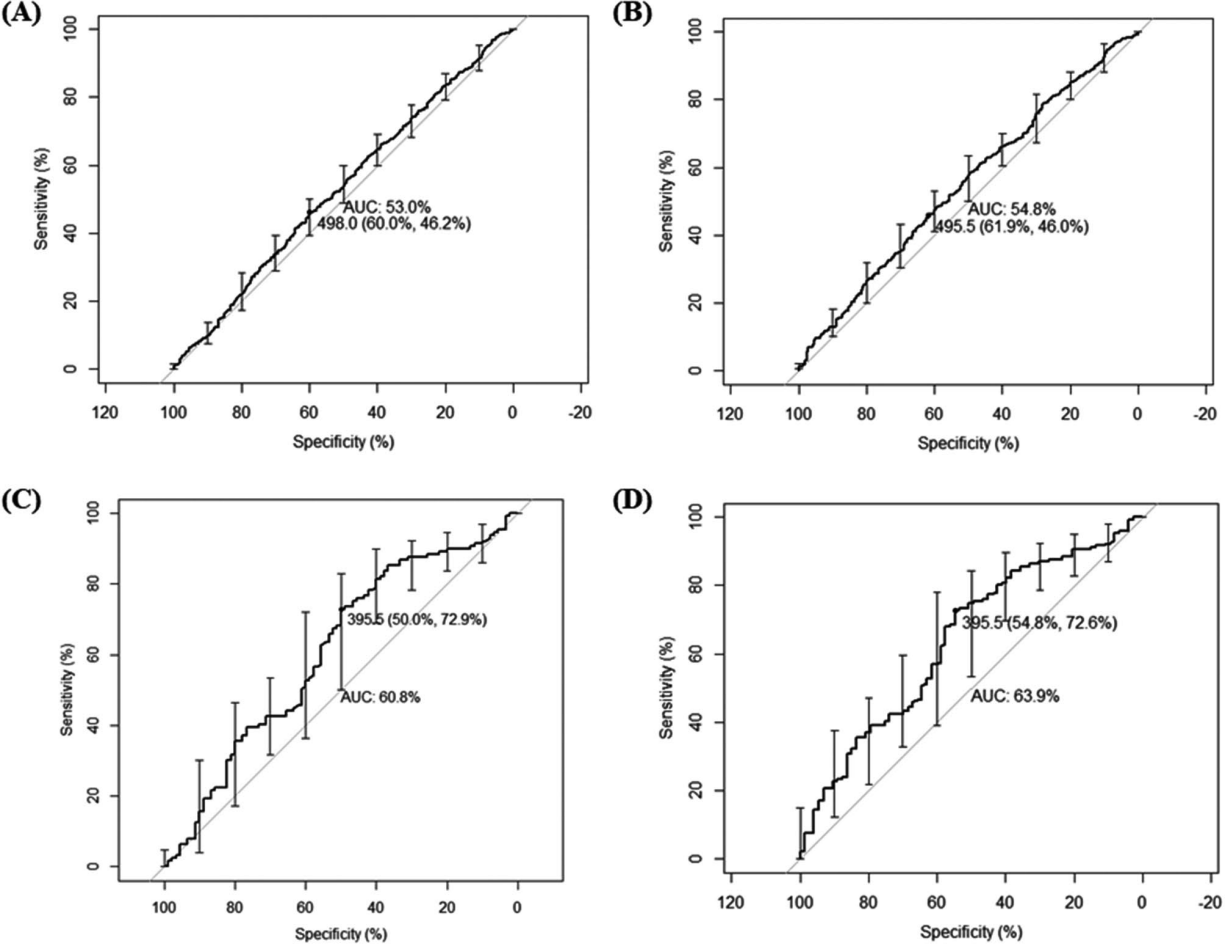
ROC curve (**A**) ROSC rates for all patients. (**B**) 24-hour survival rates for all patients. (**C**) ROSC rates for patients with initial shockable rhythm. (**D**) 24-hour survival rates for patients with initial shockable rhythm

We conducted ROC analysis to evaluate the effect of the pre-MCPR time on the prediction of ROSC and 24-hour survival. Only the pre-MCPR time could not precisely predict ROSC (area under the curve (AUC) = 53%, cutoff point = 498 s, specificity = 60%, sensitivity = 46.2%) or 24-hour survival (AUC = 54.8%, cutoff point = 495.5 s, specificity = 61.9%, sensitivity = 46%) (Fig. 3A and B). In a previous study, OHCA patients with initially shockable rhythms had significantly greater probabilities of ROSC and 24-hour survival. Thus, we conducted an additional ROC analysis for the subgroup of patients with initial rhythm. The results revealed that the pre-MCPR time was better associated with the ROSC and 24-hour survival for the patient subgroup, with AUCs of 60.8% and 63.9%, respectively, and a cutoff point of 395.5 s. (Figure 3C and D).

## Discussion

This was a retrospective observational study of OHCA patients from the NTFD EMS database in 2022. We discovered that earlier application of MCPR devices is associated with better outcomes for OHCA patients. After reviewing the records of 1680 OHCA patients, we found that young age, initial shockable rhythm, occurrence in public places and shorter transport time were key factors for better clinical outcomes, which were identical to those of the general population worldwide. We also found that early intervention with MCPR during resuscitation, including ROSC and 24-hour survival, was positively associated with better outcomes. In addition, the pre-MCPR time may be a reasonable predictor of outcomes in OHCA patients with an initially shockable cardiac rhythm, with an optimal cutoff point of 395.5 s since CPR initiation. Since prompt CPR, analysis of the patient’s initial rhythm, and timely defibrillation, if indicated, seem to be per the current protocol, early MCPR setup after the abovementioned tasks have the potential to improve outcomes, especially in the setting of small team. This is the first study concerning the impact of the timing of MCPR intervention on the outcome of OHCA patients, providing potential guidance for MCPR application in the future.

Previous studies have suggested that OHCA patients with initially shockable rhythms have a greater chance of survival, regardless of the use of manual CPR or MCPR [17, 21]. However, video review of the time spent on the MCPR setup process has rarely been performed, but we found that it might be a critical factor in our study. Our analysis revealed results similar to those of previous studies, and we also discovered that OHCA patients with shockable rhythm who received MCPR earlier tended to have better survival outcomes than those who received intervention later. According to previous studies, OHCA patients who presented with shockable rhythms may benefit from earlier delivery to the cardiac arrest center [22]. Since paramedics in the NTFD were instructed to have MCPR ready before transporting, earlier MCPR intervention may result in earlier delivery from the arrest scene to hospitals. This may explain why OHCA patients with shockable rhythms benefit from earlier MCPR application, whereas the benefit in nonshockable rhythm patients is relatively obscure.

A previous report reported that the percentage of OHCA patients receiving bystander CPR before ambulance arrival ranged from 19.1 to 79.1% worldwide [23]. Meanwhile, 54.2% of the OHCA patients in our study received bystander CPR provided by passers-by, family members, or nursing facility staff. However, we did not find an association between bystander CPR and ROSC or 24-hour survival. Owing to the current lack of TOR rules, even if arrest happens a few hours before being found, the patient might still receive complete prehospital resuscitation and transport to the hospital until death is announced. This scenario may have given rise to the relatively greater ratio of the initial nonshockable rhythm in our study than in other developed countries [24]. OHCA patients with a nonshockable rhythm benefitted less from bystander CPR [25], therefore, which may explain why bystander CPR did not contribute to survival in our study.

This study had a few limitations. First, as a retrospective observational study, our study may be subject to bias, limiting its generalizability. Second, witnessed collapse is an important factor for favorable survival outcomes, but it was not recorded by the ePCR system until 2023 and thus hindered further analysis. In addition, paramedics may decide when to apply MCPR on the basis of clinical judgments, which may include additional confounding factors that we did not consider. Finally, we could not standardize treatment after patients were admitted to various hospitals, which may have altered the outcomes.

In the future, large-scale randomized controlled trials should be conducted to establish the associations between the MCPR setup time and OHCA patient outcomes. Confounding factors related to paramedics’ decision-making strategies and whether a collapse has occurred should be considered. Patients recruited should also be stratified with different hospital levels to standardize in-hospital management for a more robust conclusion on this topic.

## Conclusion

A positive correlation was found between early MCPR intervention and treatment outcomes, including ROSC and 24-hour survival. The pre-MCPR time could predict ROSC and 24-hour survival in the subgroup of patients with initially shockable rhythms, with the optimal cutoff point being 395.5 s. Our findings may be useful for other EMS systems with similar characteristics, such as manpower shortages during the COVID-19 pandemic or tactic medicine.

## Data Availability

The data that support the findings of this study are available from the New Taipei Fire Department but restrictions apply to the availability of these data, which were used under license for the current study, and so are not publicly available. Data are however available from the authors upon reasonable request and with permission of the New Taipei Fire Department.

## Abbreviations

ACLS: Advanced cardiac life support
AED: Automated external defibrillators
AHA: American Heart Association
CCF: Chest compression fraction
CPR: Cardiopulmonary resuscitation
EMS: Emergent medical service
EMT: Emergency medical technician
ePCR: Electronic patient care reporting
MCPR: Mechanical cardiopulmonary resuscitation device
NTFD: Fire Department of New Taipei City Government
OHCA: Out-of-hospital cardiac arrest
QC: Quality control
SGA: Supraglottic airway
TOR: Termination of resuscitation
ROSC: Return of spontaneous circulation
